# Book Reviews

**Published:** 1828-05

**Authors:** 


### CRITICAL ANALYSES.

Quæ laudanda forent, et quæ culpanda, vicissim

Illa, prius, cretâ; mox hæc, carbone, *notamus.*—Persius.

*A General View of the present State of Lunatics, and Lunatic Asylums, in Great Britain and Ireland, and in some other Kingdoms.* By Sir Andrew Halliday, M.D. and K.H., Fellow of the Royal Society and College of Physicians of Edinburgh; Licentiate of the Royal College of Physicians of London, &c. &c.—8vo. pp. 101. T. and G. Underwood, London, 1828.

*Practical Observations on Insanity, and the Treatment of the Insane; addressed particularly to those who have Relatives or Friends afflicted with Mental Derangement: also Hints on the Propriety of making the Study of Mental Disorders a necessary Adjunct to Medical Education.* By W. J. late a Keeper at a Lunatic Asylum.—8vo. pp. 127. Anderson, London, 1828.

The public attention has of late been painfully directed to the subject of insanity, and the consequence is that more information has been obtained as to the condition of that unhappy class of patients in this country within these last two years, than the annals of the preceding century can afford. It may, perhaps, be said that popular prejudice has loaded with a greater share of obloquy than they deserve those establishments in which the maniac is confined, either for the purpose of security, or in the hope of obtaining a cure; but still these prejudices have been operating on the side of good feeling and right principle, and much real good has already been derived from the inquiries that Parliament has instituted.

Among the number of pamphlets that have been elicited by the interest or novelty of the subject, the two whose titles appear above were put into our hands a short time ago, and we shall devote a few pages to the consideration of their contents. Though treating of the same subject, their objects are distinct, as their title-pages announce; and we shall first introduce Sir ANDREW HALLIDAY's work to our readers' notice. It enters very slightly into the question of insanity, either in a philosophical or medical point of view, but may be consulted advantageously by those who wish to have a short account of the various lunatic asylums in England, Ireland, or on the continent, together with details as to the probable expence of maintaining such an establishment.

From the preliminary remarks upon Insanity, we extract the following passage, which describes not what was, but what we fear is too commonly the case at the present moment.

"Whether insanity proceeds from deranged organs or diseased structure, the mind, *qua* mind, is neither injured nor impaired; but not having the means of making itself known, or of giving its ideas form and utterance, except through the medium of instruments no longer fit for the purpose, we discover, in proportion to that degree of their unfitness, more or less of the phenomena that constitute madness. Through an unaccountable weakness in human nature, aided, I fear, by the representations of interested knaves, a feeling has hitherto obtained among all classes of society that a something disgraceful, nay, almost amounting to criminality, became attached to the person, and even to the family of an unhappy lunatic. The attack of the disease was, therefore, no sooner confirmed, than he was put under the charge of some heartless hireling, and hurried off to a place of concealment, where it became the interest of his keeper to have his disease made permanent. This, by seclusion or cruelty, was soon effected, when, by proper treatment, and a few soothing attentions in the bosom of his own family, it was more than probable he would speedily have recovered. And such has generally been the abandonment of friends under this affliction, that every degree of neglect and cruelty might be practised without the risk of discovery. The mystery which was made to hover round the precincts of a mad-house, was sufficient to baffle common inquiry; and the utter seclusion, so insidiously inculcated, made it next to impossible to discover the scenes of horror that took place within its walls. It would have been well for the unhappy maniac had his confinement always been entrusted to a medical practitioner, where there was some character at stake, and might be some inducement to treat him well: but in this country the law allows persons of all descriptions—men and women too, whatever may be their ignorance or incapacity,—to become the keepers of mad-houses. True it is, if they take more than one patient at a time, they must have a license for doing so; but when it is considered that the commissioners who are authorised to grant these licenses have no power to refuse them, what, I would ask, is the use of such a regulation? (P. 6.)

Our author next proceeds to relate the present state of the law respecting lunatics and lunatic asylums in England, from which it appears that, until the year 1774, no legislative enactment upon this subject existed; and this, imperfect in all its parts, and absolutely nugatory as far as the comforts of the unhappy lunatics were concerned, remained in operation until the year 1806, when a committee of the House of Commons was appointed to investigate the condition of pauper lunatics: the result of this was the passing of the Act which authorises the magistrates of the several counties to erect public asylums for their insane poor. After a lapse of seven years, this inquiry was renewed by Mr. Rose, and a Bill, founded upon the evidence then adduced, passed through the House of Commons for three or four successive years, but was uniformly rejected in the Lords. The inquiry has only been again taken up in the last session of Parliament.

Sir A. Halliday observes, that, although it is twenty years since Mr. Wynn's Act was passed, only ten asylums have been erected in the fifty-two counties of England. The number of lunatics publicly accounted for he calculates at 4782 persons; but there is, he adds, a number, if not equally great, at least nearly so, of whom the law takes no cognizance, and whose existence is only known to their relatives and friends: these are individuals placed in the care of persons who only take one patient, and who therefore have no need of a license. We leave our readers to surmise to what purposes such an unwarrantable power may be converted.

The remainder of this section is occupied with details respecting each of the county asylums; the number of patients they are capable of containing; the expenses incurred; the cost in building, and other matters worthy of notice, but which we have not the space to extract.

Our author pursues this subject with reference to these establishments in Scotland, Ireland, and the continent of Europe; from which we can only afford room for the following condensed account of the insane establishment at Antwerp. It was built twenty-five years ago, and accommodates 230 patients in separate cells, besides two infirmaries, with twenty beds in each. Each cell has its own water-closet; a stream of water runs under the building; the sexes are, of course, kept distinct, and there is a large garden and airing ground for each class. Connected with this hospital is a very singular establishment, situated at the village of Gheil, about twenty-seven miles from Antrwep, on the road to Lier and Turnhout, a place detached from other habitations, and was traditionally famous for the treatment of insanity, where the villagers were reported, in almost all cases, to have effected a cure. The founder of the present asylum caused an additional number of cottages to be built, and allotted to each a certain portion of the barren heath. These cottages were distributed to such of the community as were disposed to marry and settle, upon condition that they should receive only the convalescents from Antwerp, at a certain board, and treat them in the same manner as the other patients at Gheil: they were bound to treat these convalescents kindly, but to employ them in the cultivation of the waste lands. Here it was found that the insane recovered rapidly, and, as the fame of the villagers increased, persons of the highest rank, and even the poor, were sent to them without passing through the hospitals at all. Each patient is obliged to labour for a certain number of hours in the day, according to his strength, and, when not employed, he is allowed to walk about without restraint, and they are summoned to their homes by the bell of the village church. Scarcely any accident ever occurs, and very few ever attempt to make their escape. The author suggests the adoption of some such plan in this country.

Our readers will find also some particulars relative to the lunatic establishments in India, worthy of their attention; and in the Appendix are several documents which will be read with interest by those interested in this branch of inquiry; but we can only refer to the work itself, and now pass to a brief review of the labours of W. J.

This publication is evidently intended to be rather popular than professional, and it is written, we think, in a somewhat exaggerated style; but still it is certainly well intended, and is as evidently the fruit of much observation and experience. The author says that it was composed in the wards of a madhouse during the short spaces snatched from his duties as a keeper; and certainly there is a fund of good plain sense in many of the remarks, and many evil practices are exposed, for the truth of which we can vouch from experience.

The first chapter contains rather a verbose description of the male and female maniac: it is an evident attempt at fine writing, which the subject does not require, and is particularly unsuited to all disquisitions of a medical or philosophical nature. We therefore pass it by, and find, in the second chapter, our author asserting that the late inquiries ought not to stop at the investigation of the abuses in public asylums, and that the grievous abuses which exist in private establishments require also to be examined into, and a more efficient mode of control employed.

We perfectly agree with W. J. in the belief that numerous cases are consigned to lunatic asylums without the least necessity, and that there are hundreds of persons so incarcerated who would be much more likely to be restored to health of mind under the care of kind and considerate friends, than by being placed where every object around them must tend to excite a morbid state of feeling. (P. 9.) The following passage we give in the author's words: most of our readers will recollect a recent case in confirmation of the truth contained in it.

"It is doubtless a matter of very great convenience to many persons, that there are places to which troublesome or eccentric relatives or friends may be packed off out of the way: and it is probably at times very convenient for some people to have the management of the property of others. An unfeeling and brutal husband may exasperate a sensitive and amiable woman by his neglect and cruelty, until, with continued harassings, her mind becomes irritable and unsettled: she may be in the way on other accounts, and the husband may be anxious to get rid of her; he tells his own tale to a pliant physician, who calls to examine her, and the very questions he puts, implying doubts of her sanity, excite her to give irritable and incoherent replies, and she unconsciously seals her own doom; and the gloomy walls of a mad-house, the association with lunatics, and a strait waistcoat, soon finish the work which villany began." (P. 10.)

The remainder of this chapter is devoted to some comments upon the present mode of visiting these asylums by the commissioners under the present Act of Parliament, which visits are stated to be little more than those of courtesy: indeed, they have neither the power to remedy abuses or to refuse a license under any circumstances.

The high prices paid for the support of insane patients is next remarked upon, and some not very palatable observations made upon the tenacity with which persons who can afford to pay so handsomely are held in the grasp of their keepers.

The object of the next chapter, on *Incipient Insanity*, is to show the impropriety of consigning those in whom this dreadful malady is beginning to develope itself to the cheerless gloom of a mad-house; and our author observes of such a patient, brought up probably in the bosom of a tender and affectionate family, surrounded by the endearments of the social circle, and blessed perhaps with every comfort life can bestow,—what must be the effect on such a mind on being torn from all those ties, consigned to the society of lunatics, and to the control of strangers, whose humanity and tenderness are at any rate problematical? (P. 26.) He illustrates his position by relating instances of the ill effects of placing patients in an incipient state of insanity in contact with confirmed lunatics; but he does not dwell, we think, sufficiently upon the mischief that occasionally arises from the injudicious management of the vulgar, illiterate superintendants of these asylums; persons to whom the care of these unfortunate beings is almost entirely left, who become callous by habit, which at the same time leads them to believe that they possess some sort of indescribable knowledge of the disease, which others have not, and who are perpetually outraging the feelings and counteracting the habits of those whose sensibility and propriety of conduct they can neither appreciate nor understand. This we have seen miserably exemplified in more instances than one.

From the fifth chapter, on *Confirmed Lunacy,* we do not deem it necessary to make any extract; but, in chapter the sixth, we find our author advocating the propriety of employing a better class of people as the immediate attendants of the insane, and giving us some examples of the ill effects produced upon the unhappy patients by the substitution of force for persuasion, the usual resource of the vulgar when entrusted with power. The remedy for this does not appear to us to be sufficient to meet the evil: a mere increase of salary will never tempt a better class of persons to undertake offices of this description; but we do think that a better example on the part of the manager or superintendant (generally a female), with a proper vigilance as to this important requisite on the part of the keepers, would very much contribute to remedy the evil which our author justly complains of. Force is generally employed because, independently of the pleasure arising from supposed authority, it is falsely believed to be the quickest mode of attaining the desired end, that of obtaining the ascendancy over the patient: but the notion is a vulgar error; kindness will effect more, much more. It must be tempered with firmness; but still kindness will ever be found the surest and safest road to the confidence of the insane. A teasing, annoying, meddling line of conduct is, perhaps, as pernicious as absolute constraint and violence. In some respects, the treatment of the insane may be compared to that of the infant: you must not notice every minute deviation from a correct conduct, but whatever you do notice you must amend. If you suffer your patient to weary out your patience, and to attain a forbidden end, there is at once a period to all control and authority on your part: therefore, it behoves the attendant never to interfere unless it be necessary.

The ***Hints on the Duties of Keepers,*** the subject of the seventh chapter, are well deserving of the attention of those persons, and are very much in unison with what we have suggested above from our own observation.

We know not what to say to chapter ninth; the title is rather staggering to the medical man; it is as follows: *On the Necessity of the Medical Profession paying greater attention to the subject of Insanity.* W. J. seems to have formed strong opinions upon this point; perhaps he may be right. His opinion is, we fear, the result of what he has himself seen: at least, so we should be inclined to judge, from the relation of a case evidently of acute disease of the membranes of the brain, in which unquestionably the medical attendant made a sad mistake; but we would suggest that it is not quite fair to argue generally from a particular instance or two. For our own parts, we believe that the knowledge and management of mental diseases has advanced in as great a ratio as that of any other class of complaints, and that such mistakes as our author has recorded are very unlikely to occur frequently, if at all.

In the two concluding chapters, our author treats of two of the exciting causes of insanity, *drunkenness* and *masturbation.* Our author seems to think that a writ *de inebriato inquirendo* should issue whenever insanity decidedly proceeded from habitual inebriation, as the writ *de lunatico inquirendo* is when the person becomes deranged from any other cause. But we fear W. J. has not considered this subject very philosophically; for if a writ were to be issued to inquire into the habitual vices which make such inroads into the happiness of social life, we know not of whom the juries would be composed. With respect to the writ *de lunatico inquirendo,* we really think that, in almost all the cases of this kind, too much importance is attached to medical evidence. In the treatment of the insane, we willingly allow the medical man to be absolute, and to act without control or interference; but when the question is to be decided whether an individual is insane or not, the common sense of a well-appointed jury will elicit that fact from the testimony of the patient's servants, acquaintance, and friends, with quite as much certainty, and with less perplexity, than when the interested evidence of the professional witness is called for. Some recent examples of the very conflicting opinions publicly professed by eminent men in the profession, particularly, devoted to this branch of inquiry, amply confirm and justify these remarks. Of the latter cause, masturbation, our author treats more at large than is usual: we believe there is much truth in what he urges upon this disgusting topic, and the hint that he throws out for the guidance of the guardians of youth are well worthy of attention. We are of opinion, however, that W. J. has a little overcharged this statement; at least we hope so, when he says that full three-fourths of all recent cases, from eighteen to thirty years of age, were insane from the effects of masturbation, and that the proportion between the male and female is nearly equal. Our own experience leads us to believe that these melancholy cases very commonly terminate in idiotcy, and are not unfrequently accompanied by epilepsy.

In conclusion, we beg to repeat that, in our opinion, W. J. has performed his task very respectably: he has told many wholesome truths, which will have the more weight as coming from the pen of one who is deeply versed in all the mysteries of lunatic establishments; and, if he has exaggerated a little, that exaggeration is all on the side of humanity and right feeling.

---

*A Practical and Pathological Inquiry into the Sources and Effects of Derangements of the Digestive Organs, embracing Dejection, and some other Affections of the Mind.* By WILLIAM COOKE, Member of the Royal College of Surgeons, Secretary to the Hunterian Society, Editor of an Abridgment of Morgagni, &c. &c.—8vo. pp. 290. Longman and Co. London, 1828.

NOTWITHSTANDING the many volumes that have issued from the press upon the subjects which Mr. COOKE has selected, he is inclined to believe "there is yet ample room for other labourers, who may, at least, assist in the process without following the beaten track, or wasting time in idle discussions." We agree with him. Every observant practitioner, with the advantage of experience, may yet add to the information we possess respecting the ever-varying diseases of the digestive organs. But, as fashion bears some sway in the treatment of disease, as well as in the adornment of the person, one caution may be thrown out without impropriety. Let us be careful that, in our zeal to keep the stomach and bowels in proper order, we do not too hastily conclude that they are the original offenders, and thus neglect diseases of other parts with which the digestive organs so frequently sympathise.

"Incalculable good has resulted from directing attention to the influence of derangements of the digestive organs in producing various chronic affections; but this doctrine has been so extensively promulgated, and is now so prevalent, that the opposite truth has been somewhat overlooked. That the functions of the abdominal viscera are disturbed by injuries of the head, and by emotion of mind, are facts too common and too prominent to escape observation: but the effects of idiopathic disease in the head, or in any other viscus; or of continued pain in any part of the body; or of general debility, on the nervous system and functions of the organs in question, though not less certain, are liable to be disregarded when the mind is strongly imbued with the notion of their priority in suffering." (*Preface*, p. ii.)

"The various sources of dyspeptic symptoms" have been too frequently pointed out, and we believe are too generally understood, to require, upon the present occasion, a very lengthened notice. Congestion or chronic inflammation of the mucous membrane is not unfrequently productive of symptoms resembling those which arise from simple dyspepsia. "These affections may usually be distinguished by attentive inquiry into the history of the disease, by a careful investigation of the patient's sufferings, and by the effect of the means employed; and, undoubtedly, it is of the highest importance that they should be distinguished." (P. 4.)

In some cases, inflammation of the mucous membranes may proceed on insidiously even to ulceration, and escape detection without much attention.

"In the treatment of those diseases which arise from an inflamed state of the mucous membrane, bleeding by leeches or cupping will be one of the most available remedies; and in cases of mere congestion it may be necessary to effect a local abstraction of blood, though not generally to the same extent as in inflammation. Mercurials, when administered before depletion, I believe, are generally injurious, and even then will require to be given in the mildest forms and smallest doses." (P. 6.)

If purgatives are required in such cases, the mildest should be employed. Irritating purgatives should be avoided, as they tend to keep up the excitement in the "muciparous" texture. In one case of chronic diarrhœa, where there was reason to suppose the membrane was in the state adverted to, the author used the Sulphas Cupri, as recommended by Dr. Elliotson, with great advantage.

Under the head of *Hemorrhage from the Alimentary Canal,* an interesting case of hæmatemesis is related, but in too detailed a manner to enable us to abstract it. Although vomiting of blood, particularly when it occurs in young females, and is vicarious of suppressed menstruation, is rarely a dangerous disease, unless the general powers of the constitution are broken down by a great and long-continued discharge of blood; it occasionally happens that such cases terminate fatally. We have seen two cases, in both of which a favorable prognosis was given in consultation, and in both the patients suddenly threw up a large quantity of blood from the stomach, and quickly expired. We should therefore give a guarded opinion, even if the patient appears to be relieved of the previous symptoms of oppression at the præcordia, &c. by the discharge from the stomach. We believe we are indebted to BICHAT for the true pathology of such cases. Until his time it was generally imagined that the blood was thrown forth from some ruptured blood-vessel. He considered it to be hemorrhage from the exhalant vessels of the whole or a great part of the stomach and intestines, and his opinion was verified by dissection. In the two cases we have referred to, the internal lining of the stomach and intestines was soft, red, and pulpy, and thickly studded with minute red points. It appeared as if the membrane had been destroyed by a chemical caustic. Hæmatemesis may, however, sometimes arise from the rupture of a particular vessel.

*Gastritis and Diaphragmitis from Medicine.*—Though the stomach is capable of great resistance in health, there are states of constitution, and conditions of the organ itself, which render it extremely susceptible of irritation and inflammation. In treating subjects of this kind, great caution will therefore be necessary. A case in illustration is very minutely described. The patient was a weak, nervous, dyspeptic woman, but with a cheerful mind. She had lived beyond her income of health, by too close an attention to the duties of tuition. She had found relief from bluepill and hyosciamus, gentle aperients, and bitter infusion. Contrary to the opinion of Mr. Cooke, a stimulating medicine was determined upon in consultation, consisting of a drachm of Sp. Ammon. accompanied with Inf. Cuspariæ. Inflammation of the throat, pain and soreness in the chest, were produced, together with inflammatory action in the mucous lining of the œsophagus. Bleeding and antiphlogistic means were now required, and the patient gradually recovered from these symptoms, although she shortly afterwards fell a victim to an accumulation of disease.

In persons whose nervous system is easily deranged, we should, therefore, be very careful to adapt our treatment to their particular temperament.

Cases are given in which the common symptoms of dyspepsia were produced by chronic inflammation of the peritoneum, and from renal irritation, in which the real source of suffering was very insidious in its progress.

*Influence of the Brain on the Digestive Organs.*—When disease manifests itself in the head and abdominal viscera almost simultaneously, the affection of the former, in the present day, is usually attributed to some derangement of the latter. Two cases are related in which some ambiguity existed, and which strongly point out the necessity of not too hastily regarding the stomach as the original seat of ailment.

Mr. Cooke makes many good practical observations upon diseases of the pancreas, spleen, and liver; and illustrates his opinions by instances of each disease which have occurred in his practice.

*Influence of the Nervous System and the Mind on the Digestive Organs.*—The experiments and practical suggestions of Charles Bell, Magendie, Wilson Philip, and others, are briefly referred to, for the purpose of showing the intimate connexion which exists between the nervous system and the stomach. The various effects which in different persons are produced upon the digestive organs, and the constitution in general, by mental affections, are also briefly touched upon. Having commented upon the various sources of derangement in the organs concerned in digestion, it is very properly insisted upon that no definite mode of treatment can be laid down.

"To rely on the same medicines, to prescribe the same diet, to establish the same intervals of taking food, to enjoin the same exercises, is nothing else than empiricism. Even if we admit that the effect of the various causes may be alike, surely there must be some reference in the treatment to the cause itself, and likewise to the constitution. Sometimes bleeding will be requisite; sometimes active purging; sometimes perseverance in the mildest doses of mercurial remedies; sometimes bitters or tonics. At all times the diet must be most carefully regulated, but we should bear in mind that there are persons who cannot undergo very long intervals between the seasons of taking nourishment without being distressed, and this is particularly the case with children. I do not advocate a system of repletion, nor the ridiculous practices of parents who allow their children to be almost constantly eating; yet the digestion of children is generally more rapid than that of adults; and how often do we see some children in a school, or family, who cannot endure the same interval as their companions, but in attempting it are reduced to a state of extreme irritability and languor.

"With respect to the administration of purgatives, there are three points to which I shall just advert. There will sometimes be fatal accumulation of fæces in the intestines, when both the patient and attendants report that the bowels are freely relieved. When the obstruction arises from a mechanical cause, as hernia or contraction, great caution is necessary in the administration of purgatives. Purgatives are not unfrequently persevered in to remedy unhealthy secretions, pain, tenderness, and flatulence, solely kept up by the means employed.

"In cases of constipation we must be careful that the discharge of a loose motion does not deceive us, for this may happen without the bowels being sufficiently acted upon. We ought never to be satisfied in any serious case without careful examination with the hand, for it will frequently happen, even after fluid dejections, that a large accumulation of fæces shall exist." (P. 128.)

Mr. Cooke ventures to submit, and we believe with much propriety, that Mr. ABERNETHY has circumscribed his principles too much. We know there are many respectable writers who have denied that calomel will produce green and slimy stools. They look upon evacuations of this kind as a proof of the necessity for the further and more free use of mercurial medicines. We have no doubt that "those practitioners who redouble their forces when these appearances are detected, aggravate the symptoms they intend to allay." In the treatment of the diseases of children this error is by no means uncommon.

In the second part of the volume the author treats *on the Effects produced upon remote Parts of the Body, and upon the Mind, by Derangement of the Digestive Organs.* Numerous cases are detailed of derangement and organic disease of various parts, from an unhealthy condition of the stomach and bowels.

Mr. Cooke's work is entirely practical, and will not be consulted without advantage. From the many cases he has related, it is evident he must have extensive experience, although, if they had been less minutely detailed, the perusal of the book would certainly have been more agreeable and not less instructive.

---

*A Disquisition on the Nature and Properties of Living Animals; with an Inquiry how far our Knowledge of Anatomy and Physiology is consistent with the Belief of a Soul and a future Life, and on the Intellectual Difference between Man and Brutes.* By GEORGE WARREN, Surgeon.—8vo. pp. 144. Longman and Co. London, 1828.

As, in our opinion, physicians are not called upon to abstract their minds from their more important duties, by entering the field of metaphysical disquisition, we should have passed over the present work altogether had it been entirely confined to such speculations. We would prefer being accused of mental cowardice or inefficiency by evading a subject, which, " settled oft, remains unsettled still," rather than encourage our readers to waste their time and talent by entering upon a theme which must be unprofitable to them as practical men, although it may flatter the vanity of a subtle disputant to show that he can still say something upon a subject about which so much already has been said. But he is really wise who feels and confesses that there are limits to human reason —that Nature has surrounded man with certain boundaries, beyond which he cannot hope to advance either with success or safety.

Mr. Warren has dedicated his first chapter to a brief account of the present state of our knowledge upon the subject of life. He gives us extracts from the works of Lawrence and Sir Charles Morgan, which have been already frequently canvassed. We agree with him that the subject has been one of deep interest,—we would add, of fruitless investigation, " the philosopher's better system."

Professed writers upon animal being may be divided into three classes. The merit of each consists rather in confuting, or at least confounding, the opinions of others, than in establishing their own hypothesis. One class contend that a living principle, having an existence independent of the physical body, and endued with those faculties which constitute the mind, and being of an indestructible nature, is the life-giving principle to the animal. Another class refer every property of the living animal to organization: they deem it useless to seek for any other explanation. The third class maintain, upon moral and metaphysical grounds, that some other principle, of which their conception is very imperfect and their ideas confused, must also exist in union with matter, to explain the phenomena of mind, and to render the tenets of their faith of possible accomplishment.

" This being the present state of knowledge, it unfortunately results that he whose attention and study have been directed to the attainment of skill in the cure of diseases, has been driven, by the imperfection of science, to the necessity either of regarding animal existence in an entirely physical point of view, or of admitting the existence of some other principle than matter, with which no connexion with the organic operations could be discovered. The first admission might be the basis of science to the exclusion of any rational ground for moral principles: the latter might be the basis of moral principle to the exclusion of science. To one of these formidable results either opinion must lead, unless man can so command his thinking powers as to regard the same body in two opposite views at the same time. It is not meant by this to deny that many men, eminent in medical and surgical science, and in the practice of piety and morality, have regarded man both as a moral agent and as a physical body; but they have always been compelled to pursue in argument opposite lines of reasoning, in support of these opposed notions. And no person has ever traced any satisfactory connexion between man as a moral agent and a physical being. What connexion has ever been shown between him as a physical being and mind, has a tendency to deny his moral agency, and to make him the creature of physical necessity." (P. 14.)

Having glanced at the present state of our knowledge upon the subject, the author proceeds to investigate the nature of a living animal, and then goes on to inquire into the uses of the different organs and functions of the body, and their subserviency to life, in which he expounds and illustrates some new views, which he trusts also he satisfactorily proves.

The second chapter is entirely metaphysical, and those who have a taste for such speculations will discover in it many proofs of ingenious reasoning and mental acumen. Having shown the existence of the faculties of the soul, which must be admitted by all, whether materialists or not, Mr. Warren gives us *his* definition of life; and, as the mere attempt is one upon which every writer piques himself, we shall give his own words:

"The usual definition of life is, that 'it is the sum total of its functions.' This definition, whatever knowledge it at first appears to convey, is a mere waiving of the matter, as it may be asserted of every thing or being that it is the sum total of its qualities. My definition of life, a conclusion founded upon physiological opinions hereafter to be advanced, and to be maintained upon grounds hereafter to be jealously and suspiciously scrutinised, is, that it is a relation between an anima, or soul, and the natural laws of this material world. And the proper understanding of the mode in which this relation is accomplished, with a knowledge of the uses of the different parts of the body in effecting this, constitutes the science called physiology; a most important, and in practical medicine the most useful, branch of the science of life." (P. 34.)

Having hinted at the desponding temper of mind with which we approach such subjects, it will not be expected that we should enter upon a discussion from which we expect no conclusive result. We are bound, however, to consider every opinion offered us with attention, and to reflect upon it maturely in proportion to the difficulty with which it is surrounded. We have weighed well the arguments adduced in support of this definition, and we confess candidly that we cannot perceive any very conclusive argument, although we have frequent occasion to admire the ability and temperance with which Mr. Warren argues a point that has not seldom given rise to much unphilosophical acrimony of disputation.

We cannot enter into a detailed consideration of the train of reasoning which occupies many succeeding pages. The conclusions the author hopes he has established are—

"That in every living animal there are certain faculties or attributes to which, when considered abstractedly, may be appropriated the term 'anima' or 'soul;' that life consists of a relation between such attributes and the physical laws of the material world; that the body is the medium or instrument by which such relation is accomplished; that sensibility, muscular contractility, the organic movements, and animal combinations, depend upon the agency of electric fluid; that the ulterior use of food-taking is the supply of electric fluid; that the rapid circulation in animals is always in accordance with their degree of sensibility; that in the operation between the arterial and nervous systems, as well as in muscular contraction and organic movements, heat is evolved; and that the use of the lungs is to cool the body." (P. 86.)

In the eighth chapter we find some ingenious pathological opinions, in illustration of the author's belief of the existence of an electric principle in the living animal, and of its being the identical agent by which the properties of animals are maintained. Hunter and Abernethy have anticipated Mr. Warren in many of his observations and illustrations upon the influence which electricity exerts upon living animals. He goes, however, a step further than they have done. Mr. Abernethy expressly states "that he does not mean to affirm that electricity is life." But, if it be conceded to Mr. Warren "that the electric fluid is the *identical* agent by which the properties of animals are maintained," electricity and life must be considered as synonymous terms.

We are happy to find Mr. Warren a determined, yet temperate, opponent of the doctrines of materialism. In the last chapter, the author takes a slight comparative view of the brute and human soul. He argues that it is not unscriptural to admit a soul in all living animals, although it appears that there exists "a vast difference between the anima or soul of man, and the anima or soul of brutes; and it is evident that the soul of man is naturally, and philosophically, and peculiarly adapted for those higher contemplations and sublime hopes which religion upholds to his race."

They who have time and inclination to enter upon abstract and metaphysical questions may find some gratification in the perusal of Mr. Warren's book. The practical subjects which are touched upon are too briefly dismissed to arrest the attention of those who are more ambitious of making themselves thoroughly acquainted with the useful parts of their profession, than of distinguishing themselves as metaphysical disputants.

It would be unfair to dismiss the volume before us, without doing the author the justice of declaring that he evidently possesses considerable powers of reasoning, and very extensive information. — [illegible]

---

*Peripneumony; being the third Part of a Treatise on the Diseases of the Chest and on Mediate Auscultation.* By R. T. H. Laennec, M.D. Regius Professor of Medicine in the College of France, &c. (Continued.)

Laennec, with most modern authors, limits the application of this term to inflammation of the pulmonary substance, and divides the disease into five varieties: 1. Acute Peripneumony; 2. Partial Peripneumony and Pulmonary Abscess; 3. Gangrene of the Lungs; 4. Chronic Peripneumony; 5. Latent and Symptomatic Peripneumony. The *acute peripneumony* presents three stages or degrees: viz. *obstruction, hepatization,* and *purulent infiltration.* In the first of these the lung is externally of a livid or violet colour, more solid and heavier than natural, retaining the impressions of the fingers nearly like an œdematous limb. When cut, it is found to be injected with a frothy serous fluid; but, except in some portions where its obstruction is more solid, we can still clearly discover the natural spongy texture of the viscus.

In the second stage *(hepatization),* the lung no longer feels in any degree crepitous, but, as the name implies, resembles liver. The lungs frequently look less livid externally in this than in the preceding stage; but internally they are of a more or less deep red, forming a striking contrast with the bronchial tubes, blood-vessels, and their cellular partitions running through them. When the incised surfaces are exposed to the light in a certain direction, the pulmonary substance is perceived to have lost its cellular appearance, and presents a *granular* aspect, which our author looks upon as the "distinguishing anatomical character of inflammation of the lungs." This granular appearance becomes still more obvious when we *tear* a portion of the hepatised lung. When a lung is hepatised throughout, it appears at first sight more bulky than natural: this is occasioned merely by the lung not contracting as in the sound state. M. Laennec states that he has frequently measured the chest in cases of peripneumony, without being able to discover any dilatation of the affected side.

In the third stage, or that in which there is purulent infiltration, the substance of the lungs has some degree of hardness and the granular appearance above described, but is of a yellowish colour; the pus at first appearing in small points, which gradually combine, and the whole lung finally assumes a uniform straw or brown colour, and, when cut into, exudes a yellow, opaque, viscid matter, evidently purulent.

The three preceding stages are frequently united in various ways.

"Sometimes one lung is inflamed in the third degree through its whole extent, while the other contains only some portions affected in the first or second degree. Frequently the three degrees are found in the same lung, dividing it into as many zones, strongly contrasted, or gradually and insensibly shading one into another. The transition of one stage into another is marked by the occurrence of some points of the more advanced degree amidst a part which is only affected in the inferior degree: in this way, the transition from the first to the second stage is indicated by a red tissue, containing much frothy and bloody serosity, but still crepitous, intermixed with which are some portions of a redder colour, much more solid, not at all crepitous, containing less fluid, and granular when incised. Sometimes these indurated portions are exactly circumscribed by a pulmonary lobule; and, in children more especially, we sometimes even find dispersed, in the centre of the lungs, a certain number of lobules arrived at the stage of hepatization, while those immediately surrounding them are perfectly sound. This variety of peripneumony has in some recent works been denominated *lobular*. In these cases we may consider the inflammation as having commenced in several distinct points at the same time, and, being interrupted in its course by the treatment or some other cause, has not extended to the rest of the lung, or, having extended to it in a slight degree, has terminated in resolution before death. We may convince ourselves of the correctness of this opinion by examining different lungs in different stages of resolution from inflammation. The transition from the second to the third stage is marked by the existence of yellowish, irregular, uncircumscribed spots, amid a portion of lung inflamed in the second degree, the one colour passing insensibly into the other. It is this state of lung which, by the union of the two colours mentioned with the black or grey stripes produced by the black pulmonary matter, presents an appearance extremely like a piece of granite composed of red and yellow felspar, grey quartz, and black mica.

"The lower parts of the lungs are those most commonly occupied by peripneumony; and, when the disease involves the whole viscus, it is almost always in the inferior part that it commences.

When the three degrees of inflammation exist in different parts of the same lung, the site of the more advanced stage is usually in the same inferior portion. It is much more uncommon to find the inflammation confined to the upper lobe. It is not so unusual, however, to find a part of the centre of the lung inflamed, while the superficial portion is in a sound state. We never find the whole of both lungs inflamed in the third, or even second degree; and this for obvious reasons; since an obstruction of this kind could not take place instantaneously, and must render respiration quite impossible. But it is by no means uncommon to meet with cases in which one whole lung, and more than half the other, are quite impervious to air. On the other hand, we find death take place before the obstruction has reached the fourth part of the organs of respiration; a fact which, as well as many others, proves that death is frequently occasioned much more by the exhaustion of the vital principle than by the extent or intensity of the organic alteration. The right lung is more frequently affected than the left, not only in cases of pneumonia, but in almost all the other morbid affections to which these organs are subject. This fact has been long noticed by practitioners and medical writers, and is noticed among others by Morgagni." (P. 198.)

*Abscess.*—A real collection of pus in the substance of the lungs is very uncommon; our author not having met with it more than five or six times among several hundred dissections. This is very different from the statements of other pathologists, especially in this country, and is to be accounted for by the different meaning attached to the word abscess.

*Treatment of Peripneumony.*—The treatment of every disease is undoubtedly the most important part of its history, and we therefore pass by the "signs and symptoms" of peripneumony to arrive at this part of the subject. Laennec justly observes that, from the time of Hippocrates to the present day, BLOOD-LETTING has been looked upon as the great remedy in inflammation of the lungs. But, while this general principle has been admitted, the same uniformity has not prevailed with regard to the extent to which it ought to be carried, the period of the disease in which it is most advantageous, and other minor circumstances. Formerly the practice was to bleed but once, viz. at the onset of the disease, and until syncope was produced. This method our author says is still frequently adopted in England: a statement, however, not strictly correct, because, although we frequently bleed *ad deliquium*, we do not content ourselves with one blood-letting; unless, indeed, in those rare cases in which the first evacuation cuts short the inflammation. The extent to which the abstraction of blood may be carried at once varies in almost every patient: perhaps twenty-four ounces may be about the average; but the intelligent translator of the work before us mentions that he has known a man bled in an attack of fever to eighty-four ounces at once, "without suffering syncope, or any ill effect, except great disorder of the circulation for some hours afterwards:" a very remarkable fact certainly, and a practice by no means to be copied. Except that our author rather underrates both the actual and proper extent of the blood-letting, the following remarks are apposite:

"The practice most commonly followed at present over the whole of Europe is, in the beginning of the disease, to bleed to the extent of from eight to sixteen ounces, and to repeat the operation daily, and sometimes even twice a day, if the inflammatory symptoms do not give way, or if, after being subdued for a few hours, they return with fresh violence. After the first five or six days, the bleedings are repeated after longer intervals, and soon cease altogether, except in cases where they are strongly indicated by the renewed strength of the pulse, oppression, and fever. Much importance was formerly attached to the particular vein to be opened, the preference being given to that of the affected side. At present this is almost universally acknowledged to be a matter of complete indifference.

"In all these cases, and indeed in every case whatsoever, the more feeble the pulse is, the less indication is there for venesection. At the same time, it is well known to every practitioner that this feebleness is sometimes only apparent, and that bleeding will render the pulse both stronger and fuller. To discriminate the false from the real feebleness of pulse, requires the tact of an experienced practitioner; and, unfortunately, the most expert are in this often deceived. In cases of this kind, the use of the stethoscope will tend greatly to remove our doubts, as will be seen when we come to treat of the exploration of the heart's action. At present I shall only observe, that, whenever the pulsations of the heart are (proportionally) much stronger than those of the arteries, we may bleed without fear, and with the certainty of finding the pulse rise; but that, if the heart and pulse are both weak, the detraction of blood will almost always occasion complete prostration of strength. I have, nevertheless, observed in some cases, but very rarely, that a small bleeding, even in such circumstances, has succeeded in restoring the energy of the circulation; and this has been when the debility depended on cerebral congestion." (P. 241, &c.)

*Blisters*, and other counter-irritants, hold but a low rank in the estimation of our author; at least, in the acute stages of the disease.

*Evacuants.*—Laennec confines himself to keeping the bowels open by means of clysters and gentle laxatives.

*Tonics.*—We have never seen inflammation of the lungs, properly so called, in which tonics were admissible; but, when the inflammation has ended in suppuration or gangrene, or those sloughing abscesses which are more frequent than the latter, we can easily imagine those remedies of service. Laennec observes of tonics—

"These, and especially bark, are often very useful in the peripneumonies of old persons and debilitated and cachectic subjects, especially towards the termination of the disease, when, after the suppurative stage, the fever passes off, and resolution goes on very slowly. In the same circumstances the ancients recommended wine; a practice which I have myself sometimes followed with success. We sometimes even meet with epidemic peripneumonies in which blood-letting is constantly hurtful, and the bark beneficial in every stage of the disease. This fact, which cannot be denied, was frequently witnessed, particularly in Germany, towards the close of the last century; and there is no doubt that Brown's theory was indebted to this medical constitution for a portion of the fame it obtained in that country. Numerous examples of the same kind are recorded in the old Journal de Medicine; and I have myself met with many, particularly in the epidemic among the troops in 1814, already mentioned. In gangrene of the lungs, cinchona is the best remedy. I have used it successfully, even in cases where the hepatization around the eschar was very extensive; and have sometimes even combined wine and opium with it, when the violence of the inflammatory symptoms had begun to subside. To be effectual it must be given to the extent of an ounce of the powder, or an equivalent portion of the extract, daily. In several cases I have continued to give the sulphate of quinine for more than a month, to the extent of eighteen grains in the twenty-four hours." (P. 246.)

*Tartar Emetic.*—The most interesting part of Laennec's observations relate to his experience with this remedy: he thus describes his mode of using it, and the results:

"As soon as I recognise the existence of the pneumonia, if the patient is in a state to bear venesection, I direct from eight to sixteen ounces of blood to be taken from the arm. I very rarely repeat the bleeding, except in the case of patients affected with disease of the heart, or threatened with apoplexy, or some other internal congestion. More than once I have even effected very rapid cures of intense peripneumonies without bleeding at all; but, in common, I do not think it right to deprive myself of a means so powerful as venesection, except in cachectic or debilitated subjects. In this respect M. Rasori does the same. I regard blood-letting as a means of allaying for a time the violence of the inflammatory action, and giving time for the emetic tartar to act. Immediately after bleeding I give one grain of the tartar emetic, dissolved in two ounces and a half of cold weak infusion of orange leaf, sweetened with half an ounce of syrup of marsh-mallows or orange-flowers; and this I repeat every second hour for six times; after which I leave the patient quiet for seven or eight hours, if the symptoms are not urgent, or if he experiences any inclination to sleep. But if the pneumonia has already made progress, or if the oppression is great, or the head affected, or if both lungs or one whole lung is attacked, I continue the medicine uninterruptedly, in the same dose and after the same intervals, until there is an amendment, not only in the symptoms, but indicated also by the stethoscopic signs. Sometimes even, particularly when most of the above-mentioned unfavorable symptoms are combined, I increase the dose of the tartar emetic to a grain and a half, two grains, or even two grains and a half, without increasing the quantity of the vehicle. Many patients bear the medicine without being either vomited or purged. Others, and indeed the greater number, vomit twice or thrice, and have five or six stools the first day; on the following days they have only slight evacuations, and often indeed have none at all. When once tolerance of the medicine (to use the expression of Rasori) is established, it even very frequently happens that the patients are so much constipated as to require clysters to open the body. When the evacuations are continued to the second day, or when there is reason to fear on the first that the medicine will be borne with difficulty, I add to the six doses, to be taken in twenty-four hours, one or two ounces of the syrup of poppies. This combination is in opposition to the theoretical notions of Rasori and Tommasini, but has been proved to me by experience to be very useful. In general, the effect of tartar emetic is never more rapid or more efficient than when it gives rise to no evacuation: sometimes, however, its salutary operation is accompanied by a general perspiration. Although copious purging and frequent vomiting are by no means desirable, on account of the debility and the hurtful irritation of the intestinal canal which they may occasion, I have obtained remarkable cures in cases in which such evacuations had been very copious. I have met with very few cases of pneumonia where the patient could not bear the emetic tartar; and the few I have met with occurred in my earliest trials; insomuch that this result now appears to me to be attributable rather to the inexperience and want of confidence of the physician, than to the practice. I now frequently find that a patient who bears only moderately six grains with the syrup of poppies, will bear nine perfectly well on the following day. At the end of twenty-four or forty-eight hours at most, frequently even after two or three hours, we perceive a marked improvement in all the symptoms. And sometimes even we find patients, who seemed doomed to certain death, out of all danger after the lapse of a few hours only, without having ever experienced any crisis, any evacuation, or indeed any other obvious change but the rapid and progressive amelioration of all the symptoms. In such cases the stethoscope at once accounts for the sudden improvement, by exhibiting to us all the signs of the resolution of the inflammation. These striking results may be obtained at any stage of the disease, even after a great portion of the lung has undergone the purulent infiltration. As soon as we have obtained some amelioration, although but slight, we may be assured that the continuation of the remedy will effect complete resolution of the disease, without any fresh relapse; and it is in regard to this point more particularly that the greatest practical difference between the emetic tartar and blood-letting consists. By the latter measure, we almost always obtain a diminution of the fever, of the oppression and the bloody expectoration, so as to leave both the patient and the attendants to believe that recovery is about to take place: after a few hours, however, the unfavorable symptoms return with fresh vigor; and the same scene is renewed, often five or six times, after as many successive venesections. On the other hand, I can state that I have never witnessed these renewed attacks under the use of the tartar emetic. In these cases we observe only, in the progress towards convalescence, occasional stoppages. And this is more particularly the case in respect of the stethoscopic signs; as we find that, between the period when the patient experiences a return of his appetite and strength, and fancies himself quite cured, and the period at which the stethoscope ceases to give any indication of pulmonary engorgement, more time frequently elapses than between the invasion of the disease and the beginning of the convalescence. It is necessary to observe, however, that this remark is still more frequently applicable to the disease when treated by blood-letting; and, moreover, that the patients subjected to the antimonial method never experience the long and excessive debility which too often accompanies the convalescence of those who had been treated by repeated venesections.

"The best way of appreciating any particular mode of treatment is by its results. I am sorry to say that I only began last year to keep an exact account of the results of mine by the tartar emetic; but I can affirm that I have no recollection of death from acute pneumonia in any case where this medicine had been taken long enough for its effects to be experienced. I have only witnessed a few fatal terminations where the case was a slight peripneumony complicated with severe pleurisy. (We shall find, when we come to treat of the latter disease, that, after the first stage, the emetic tartar has little effect in it.) I have also lost some patients who, besides the pneumonia, were affected with cancer, phthisis, disease of the heart, &c.; and these are the cases where I had an opportunity of observing the different degrees of resolution in this disease. Finally, I have lost some who were brought to the hospital moribund, and who sunk before they had taken more than two or three grains of the remedy. In the year 1824, at the clinic of the Faculty of Medicine, I treated by the tartar emetic twenty-eight cases of pneumonia, either simple or complicated with slight pleuritic effusion. Most of these cases were very severe, yet they were all cured, with the single exception of a cachectic old man of seventy, who took but little of the medicine, because he bore it badly. During the present year (1825) I have treated thirty-four cases in the same manner. Of these, five died: but of this number two women, one aged fifty-nine and the other sixty-nine, were brought to the hospital moribund, and sunk before they had taken more than two or three doses of the emetic tartar; a third died of disease of the heart when convalescent from the pneumonia; and a fourth fell a victim to chronic pleurisy, also in the period of resolution of a sub-acute peripneumony. These two last cases will be detailed hereafter; the one at the end of the present chapter, the other in the section on Pleuro-pneumonia. The fifth case was that of a man, seventy-two years of age, who died of cerebral congestion on the tenth day of the disease. Of these five cases, then, the two first cannot be adduced in either way as instances of the effect of this remedy; and the two next are proofs of its efficacy in pneumonia, rather than the contrary. The result, therefore, of the whole is, that of fifty-seven cases of pneumonia treated by the tartar emetic, only two individuals, both upwards of seventy, died of this disease conjoined with cerebral congestion; that is, a little less than one in twenty-eight." (P. 250.)

---

*Report made by the Faculty of Medicine of Paris, in answer to an Application from the Prefect of Police, respecting the presumed Violation of a Child of Fourteen Months old.* (*Repertoire d'Anatomie,* tome iv. No. 4.)*

THE question submitted to the decision of the Faculté de Médecine by the legal authorities was, whether it was possible that an infant of such an age could have been violated. Their opinion was also requested upon the facts stated by a pupil of the Hôpital Beaujon, who reported that "the hymen was torn, and that it appeared to have been recently ruptured." As, in a legal point of view, any forcible attempt to perpetrate such a crime is as criminal as its consummation, the Faculty confined themselves to one point of inquiry, namely, to ascertain whether the appearances of the organs of generation justified the suspicion that violence had been inflicted. The certificate of the surgeon who first examined the infant stated that the hymen was ruptured, that the rupture *appeared* to be recent, and that the labia *appeared* to be inflamed. The commissioners observe, that every year, particularly during cold and damp weather, female infants are taken to the hospital with a purulent, and sometimes bloody, discharge

* This article was intended for the COLLECTANEA; by a mistake of the printer it has been introduced here.

from the vagina, accompanied by tumefaction of the parts, ecchymosis of the labia, and "solution de continuité de l'hymen." The children in this state are usually feverish, and out of health in other respects. Many of these little patients have been presented at the consultations under the impression that some improper violence had been inflicted. But a variety of circumstances tended to prove that there was no foundation for such suspicions. Several of the infants were still at the breast, and had never been left by their mothers; and the number of such cases appeared to prove the existence of some general cause which had acted simultaneously upon many different children.

In reference to the facts stated in the certificate, it was observed, that the hymen may be ruptured in many different ways, and that it cannot be determined by any examination *how* the rupture has been caused. The inflammatory symptoms, ecchymosis of the labia, and discharge from the vagina, cannot, without some collateral evidence, be asserted to arise from any criminal attempt. The report from the Faculté de Médecine, therefore, merely certified that the child laboured under a "catarrh" of the external genital organs. They did not venture to give any opinion as to the cause of the complaint.

In more than one instance we have known much anxiety excited by the appearance of symptoms in young female children, resembling mild gonorrhœa. The brief relation of a case which occurred a short time ago in our own practice, will show the necessity for much caution on the part of the practitioner. A girl, nine years of age, was attacked with the symptoms above enumerated: discharge from the vagina, swelling of the labia, and inflammation of the parts. In this state she was presented to a very young surgeon, who injudiciously declared that the child must have been the subject of some improper violence. She was repeatedly and severely questioned by her mother, and at length an accusation was extorted from her against a young man residing in the same house. He fortunately, however, was known to have been absent on the particular days mentioned by the child. Being still urged by her mother, she afterwards told the same story of two or three other men; and, when we questioned her upon the subject, she confessed that no part of her story was true, but that she believed her mother "wanted her to find out somebody."

We were consulted in a similar case some years ago, in which a criminal prosecution was about to be commenced against a man upon very slender suspicion. In this instance the child had afterwards frequent attacks of the same kind at very distant intervals. Cooling lotions and mild aperients have always succeeded in removing the inflammation and discharge.

